# Effect of Tongxinluo on Nephrin Expression via Inhibition of Notch1/Snail Pathway in Diabetic Rats

**DOI:** 10.1155/2015/424193

**Published:** 2015-08-31

**Authors:** Fangqiang Cui, Dawei Zou, Yanbin Gao, Na Zhang, Jinyang Wang, Liping Xu, Jianguo Geng, Jiaoyang Li, Shengnan Zhou, Xinyao Wang

**Affiliations:** ^1^Beijing Key Lab of TCM Collateral Disease Theory Research, School of Traditional Chinese Medicine, Capital Medical University, No. 10, Youanmenwai, Xitoutiao, Fengtai District, Beijing 100069, China; ^2^Department of Endocrinology, Gansu Provincial People's Hospital, 204 West Donggang Road, Lanzhou, Gansu 730000, China

## Abstract

Podocyte injury is an important mechanism of diabetic nephropathy (DN). Accumulating evidence suggests that nephrin expression is decreased in podocyte in DN. Moreover, it has been demonstrated that tongxinluo (TXL) can ameliorate renal structure disruption and dysfunction in DN. However, the effect of TXL on podocyte injury in DN and its molecular mechanism is unclear. In order to explore the effect of TXL on podocyte injury and its molecular mechanism in DN, our in vivo and in vitro studies were performed. Our results showed that TXL increased nephrin expression in diabetic rats and in high glucose cultured podocyte. Meanwhile, TXL decreased ICN1 (the intracellular domain of notch), HES1, and snail expression in podocyte in vivo and in vitro. More importantly, we found that TXL protected podocyte from injury in DN. The results demonstrated that TXL inhibited the activation of notch1/snail pathway and increased nephrin expression, which may be a mechanism of protecting effect on podocyte injury in DN.

## 1. Introduction

Diabetic nephropathy (DN) has become the leading cause of end stage renal disease. As we know, microalbuminuria is an early clinical hallmark of DN. The presence of albuminuria indicates the injury of the glomerular filtration barrier (GFB), of which podocyte is an important component [1]. Accumulate evidence has shown that podocyte loss and injury are an important mechanism of DN [2–4].

Nephrin is a signature molecule of podocyte [5]. Nephrin plays an important role in regulating many pivotal functions of podocyte, including signal transduction and cytoskeletal reorganization. Decreased expression of nephrin is a good marker of podocyte injury. It has been demonstrated that nephrin expression is significantly decreased in DN [6–9].

Evidence is available that notch pathway is a common pathway of podocyte injury [10]. Waters et al. [11] demonstrate that notch pathway activation can lead to podocyte injury in vivo and in vitro. It has been demonstrated that the activation of notch pathway can downregulate nephrin expression in previous studies [11, 12]. Moreover, Gagliardini et al. demonstrate that the notch1 and snail signaling is persistently activated in podocyte in DN, which is a major molecular mechanism of decreased nephrin expression [13]. Thus, increased ICN1 and snail expression of podocyte can decrease nephrin expression and then induce podocyte injury in DN. The regulation of ICN1 and snail expression has been an important target for protecting podocyte against injury in DN.

TXL, which is a new kind of Chinese herbal compound, consists of a group of herbal medicines, such as* Panax ginseng* and* Paeonia lactiflora* Pallas. TXL has been widely used in clinical practice in China over recent years. It has been demonstrated that TXL is beneficial for heart disease and cerebral disease [14, 15]. Many previous studies have been performed to explore the mechanism of TXL on heart disease and cerebral disease. It has been found that TXL can protect against pressure overload-induced heart failure by activating VEGF/Akt/eNOS pathway [16]. Moreover, Cui et al. have demonstrated that TXL protects cardiac microvascular endothelial cells through increasing autophagy and activating MEK/ERK pathway [17]. Liu et al. found that TXL regulates extensive blood-brain barrier disruption in ischemic stroke via its anti-inflammatory effect [18]. Moreover, positive therapeutic efficacy of TXL in patients with DN was observed in our clinical practice. It has been demonstrated that ginsenoside Rg1, a major active component of TXL, can protect podocyte from injury [19, 20]. More importantly, our previous study has found that TXL can ameliorate renal structure and function through inhibiting renal tubular epithelial-to-mesenchymal transition in diabetic rats [21]. However, the effect of TXL on podocyte injury and its mechanism is unclear.

In the present study, TXL decreased ICN1, HES1, and snail expression and increased nephrin expression in vivo and in vitro. More importantly, TXL had protecting effect on podocyte injury in diabetic rats and in high glucose cultured podocyte. Taken together, our results showed that TXL inhibited the activation of notch1/snail pathway and increased nephrin expression, which may be a mechanism of protecting effect on podocyte injury.

## 2. Materials and Methods

### 2.1. Preparation of TXL

TXL superfine powder was provided by Shijiazhuang Yiling pharmaceutical Co. (Hebei, China). The herbal drugs of TXL were seriously authenticated and standardized through their marker compounds according to the* Chinese Pharmacopoeia* (2005). The species, origin, harvest time, medicinal parts, and concoction methods for each component were strictly standardized. The components of TXL were ground to superfine powder with a diameter ⩽10 *μ*m by a Micronizer. Moreover, the security of TXL has been approved by the State Food and Drug Administration in China. In our in vivo study, TXL superfine powder was dissolved in aquadistillate and intragastrically administered at 60 mg/kg for DN rats each day. In our in vitro study, the TXL superfine powder was dissolved in serum-free DMEM/low glucose medium and sonicated for 1 hour. Then the TXL solution was centrifuged at 3,500 rpm for 10 min and the supernatant was filtrated by a 0.22-*μ*m micropore filter. In order to calculate practical volume of dissolved TXL superfine powder, the precipitate was heated and dried. The final concentration of TXL superfine powder solution is 2000 *μ*g/mL [22]. In our in vitro study, the TXL superfine powder solution was diluted by DMEM/low glucose medium for subsequent use.

### 2.2. Animals

The experiments were in accordance with the National Institutes of Health Guide for the Care and Use of Laboratory Animals and were approved by the Institutional Animal Care and Use Committee at Capital Medical University. Male Sprague-Dawley rats, aged 8 weeks and weighing 180–200 g, were purchased from the Chinese Academy of Medical Sciences (Beijing, China). All rats were maintained in a conventional environment with a regular 12 h light/dark cycle and 24 ± 1°C temperature. The diabetic rats were induced by intraperitoneally injecting STZ at 60 mg/kg. A total of 12 rats were injected with an equal volume of vehicle (0.1 M citrate buffer, pH 4.5) as control. Forty-eight hours after injection, tail vein blood was collected and serum glucose was detected in all rats. The diabetic rat model was considered to be successful when its serum glucose ≥16.7 mmol/L. The diabetic rats were randomly divided into two groups: diabetic nephropathy group (DN group, *n* = 12) and DN with TXL group (TXL group, *n* = 12). The rats injected with vehicle were as normal control group (NC group, *n* = 12). The rats of TXL group were treated with TXL solution (TXL superfine powder, 0.75 g·kg^−1^·d^−1^, gavage) and the rats of NC group and DN group were treated with an equal volume of vehicle (normal saline, NS, gavage). All rats were provided with free access to food and water throughout the experiment. The rats of the three groups were observed for 12 weeks. At the end of 0, 4, 8, and 12 weeks, serum glucose and urinary albumin excretion (UAE) were detected. At the end of 12 weeks after STZ, all rats were sacrificed and renal cortex was collected for electron microscope observation and purposed experiments.

### 2.3. Cell Culture

The conditionally immortalized mouse podocyte line was obtained from the National Platform of Experimental Cell Resources for Sci-Tech. Cell was cultured under permissive conditions at 33°C in DMEM/low glucose (Hyclone) medium supplemented with 10% fetal bovine serum (Excell) and recombinant IFN-*γ* (PEPROTECH) for proliferation. In order to induce differentiation, podocytes were grown under nonpermissive conditions at 37°C in DMEM/low glucose medium without recombinant IFN-*γ*. When they grew to about 80% confluence, podocytes were maintained in serum-free conditions for 24 h. To detect the optimum concentration of TXL, podocytes were treated with different concentrations of TXL (10, 25, 50, 100, and 150 *μ*g/mL) for 24 h. The podocytes were divided into three groups: normal control group (NC group, DMEM containing 5.5 mmol/L glucose + 24.5 mmol/L mannitol), high glucose group (HG group, DMEM containing 5.5 mmol/L glucose + 24.5 mmol/L glucose), and TXL group (DMEM containing 30 mmol/L glucose + 100 *μ*g/mL). The podocytes of three groups were treated for 24 h and then were used for purposed experiments.

*MTT Assay*. Cells were cultured in a 96-well plate at 5000 cells/well. After incubation with serum-free DMEM, podocytes were treated with different concentrations of TXL (10, 25, 50, 100, 150, and 200 *μ*g/mL) for 24 h. After that, cells were cultured with MTT solution at a concentration of 0.5 mg/mL for 4 h. The MTT solution was removed and DMSO was added to dissolve the purple crystals of formazan. The optical density (OD) was detected by a spectrophotometer at 570 nm.

### 2.4. Electron Microscopy

Renal cortexes were fixed with 2% glutaraldehyde in 0.1 mol/L phosphate buffer at 4°C for 120 min. They were then sectioned into ultrathin slices and were double stained with 4% uranyl acetate and lead citrate. The ultrathin slices were observed by a Hitachi 7100 transmission electron microscope (Hitachi High Technologies, Tokyo, Japan).

### 2.5. Western Blot Analysis

Renal cortexes and treated cells were collected and lysed as described previously [23]. Equal amounts of protein (20 *µ*g per lane) were separated by electrophoresis through 10% SDS-PAGE and then transferred to polyvinylidene difluoride membranes. After blocking with 5% nonfat dry milk in PBS + 0.05% Tween 20, the membranes were treated with primary antibody, washed, and then incubated with peroxidase secondary antibody. Antibodies and dilutions included the following: rabbit monoclonal to nephrin antibody (1 : 2000, Sigma), goat polyclonal to snail antibody (1 : 500, Everest Biotech), rabbit polyclonal to notch1-cleaved-val1744 antibody (1 : 2000 Abcam), and mice monoclonal to GAPDH antibody (1 : 20000). The blots were visualized with LumiGLO reagent and peroxide, followed by exposure to X-ray film. Western blot analyses were performed at least in triplicate.

### 2.6. Immunohistochemistry

Renal tissue sections (4 *μ*m) were used to perform immunohistochemical staining for nephrin and snail. After antigen retrieval, the sections were incubated with the primary antibodies of nephrin (Abcam, 1 : 100) and snail (Everest, 1 : 50). Then renal tissues were incubated with species-specific secondary antibodies and diaminobenzidine. After that, the sections were counterstained with hematoxylin and observed with fluorescent microscope. The semiquantitative analyses for immunohistochemistry study were performed using Image-Pro Plus 6.0 software. Twenty high-power microscope fields of each group were randomly selected, and their optical density (OD) was detected. Mean optical density (MOD) of each group was calculated and then used for statistical analysis.

### 2.7. Immunofluorescence

Cells were cultured on the cover glasses in 24-well plates. When grown to about 80% confluence, cells were fixed with 4% paraformaldehyde for 30 min. After blocking the nonspecific binding sites, cells were incubated with primary antibodies at 4°C overnight. After washing in PBS, cells were incubated with secondary antibodies at room temperature for 2 h, followed by counterstaining with DAPI. Cells were observed under confocal microscope (Leica TCS SP5 MP, Leica, Heidelberg, Germany). Antibodies and dilutions were as follows: rabbit polyclonal to notch1-cleaved-val1744 antibody (1 : 200, Abcam) and goat polyclonal to snail antibody (1 : 200, Everest Biotech).

### 2.8. Hoechst 33258 Staining

Cells were cultured on the cover glasses in 24-well plates. When grown to about 80% confluence, cells were fixed with 4% paraformaldehyde for 30 min. After that, cells were incubated with Hoechst 33258 for 30 min at room temperature. Cells were observed under fluorescent microscope. Apoptotic cells were identified by nuclear condensation and/or fragmentation. The number of apoptotic cells was counted in 10 random fields per group.

### 2.9. Phalloidin Staining

Cells were cultured on the cover glasses in 24-well plates. When grown to about 80% confluence, cells were fixed with 4% paraformaldehyde for 30 min. After that, cells were incubated with phalloidin for 30 min at room temperature. After washing in PBS, cells were counterstained with DAPI. Cells were observed under a confocal microscope (Leica TCS SP5 MP, Leica, Heidelberg, Germany).

### 2.10. Real-Time RT-PCR

Total RNA was isolated using the TRIzol reagent (Invitrogen) according to the manufacturer's instructions. Then RNA was reverse transcribed into cDNAs by the SuperScript RT kit (Invitrogen). Relative mRNA levels were examined using SYBR Green real-time quantitative reverse transcription-PCR (qRT-PCR) (Applied Biosystems) and were calculated by the 2^−ΔΔCt^ method. The sequences of primers are the following: mice nephrin: forward primer, 5′-CCCAACACTGGAAGAGGTGT-3′, reverse primer, 5′-CTGGTCGTAGATTCCCCTTG-3′; rat HES1: forward primer, 5′-CAAACCAAAGACAGCCTCTG-3′, reverse primer, 5′-ATGCCGGGAGCTATC-TTTCT-3′; mice HES1: forward primer, 5′-CACGACACCGGACAAACCA-3′, reverse primer, 5′-GCCGGGAGCTATCTTTCTTAAGTG-3′; mice snail: forward primer, 5′-AGCCCAACTATAGCGAGCTG-3′, reverse primer, 5′-CCAGGAGAGA-GTCCCAGATG-3′. All RT-PCRs were performed in triplicate and the data were presented as mean ± SD.

### 2.11. Statistical Analysis

Data were presented as mean ± SEM. Statistical analyses were performed by one-way ANOVA followed by the Bonferroni multiple comparison test (for comparison of more than 2 groups) or Student's *t*-test (for comparison of 2 groups). *P* < 0.05 was considered statistically significant.

## 3. Results

### 3.1. The Cytotoxicity and Optimum Concentration of TXL for Cultured Podocyte

**Figure 1:**
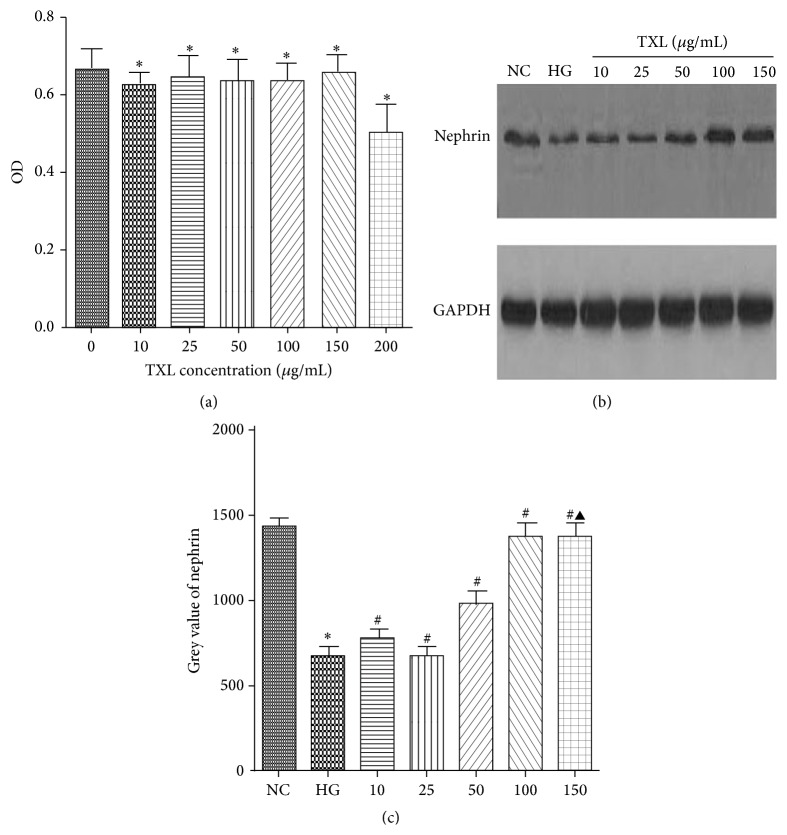
The cytotoxicity and optimum concentration of TXL for cultured podocyte. (a) Comparison of OD in cultured podocyte. The result showed that, compared with control, the OD had not significantly changed with TXL at the concentrations of 10, 25, 50, 100, and 150 *μ*g/mL (*P* > 0.05); the OD was significantly decreased with TXL at the concentration of 200 *μ*g/mL (*P* < 0.05). (b) Representative band of nephrin protein by western blot in cultured podocyte. (c) Comparison of the grey value of nephrin protein in cultured podocyte (*n* = 3). The result showed that nephrin protein was statistically significantly decreased in HG group compared with NC group (*P* < 0.05). TXL at the concentrations of 10 *μ*g/mL and 25 *μ*g/mL had no effect on nephrin expression compared with HG group (*P* > 0.05). Nephrin expression began to be increased in high glucose cultured podocyte when TXL concentration was added to 50 *μ*g/mL (*P* < 0.05). Nephrin expression was peaked at TXL concentration of 100 *μ*g/mL (*P* < 0.05). When TXL concentration was added to 150 *μ*g/mL, nephrin expression had no change compared with the group treated with 100 *μ*g/mL (*P* > 0.05). ^*∗*^ *P* < 0.05 versus NG. ^#^ *P* < 0.05 versus HG. ^▲^ *P* < 0.05 versus TXL (100 *μ*g/mL).

To detect the cytotoxicity of TXL on podocyte, MTT assay was performed in our study. Our results showed that, compared with control, TXL at 10, 25, 50, 100, and 150 *μ*g/mL has no cytotoxicity on podocyte. However, cell viability was significantly decreased when it was incubated with TXL at 200 *μ*g/mL. To determine the optimum concentration of TXL, nephrin protein expression was detected by western blot in cultured podocyte treated with different concentrations of TXL (10, 25, 50, 100, and 150 *μ*g/mL). Our results showed that HG repressed nephrin expression and TXL increased nephrin expression in high glucose cultured podocyte. More importantly, we found that TXL at 100 *μ*g/mL had the optimum effect on nephrin expression in high glucose cultured podocyte (Figure 1).

### 3.2. TXL Increased Nephrin Expression In Vivo and In Vitro

**Figure 2:**
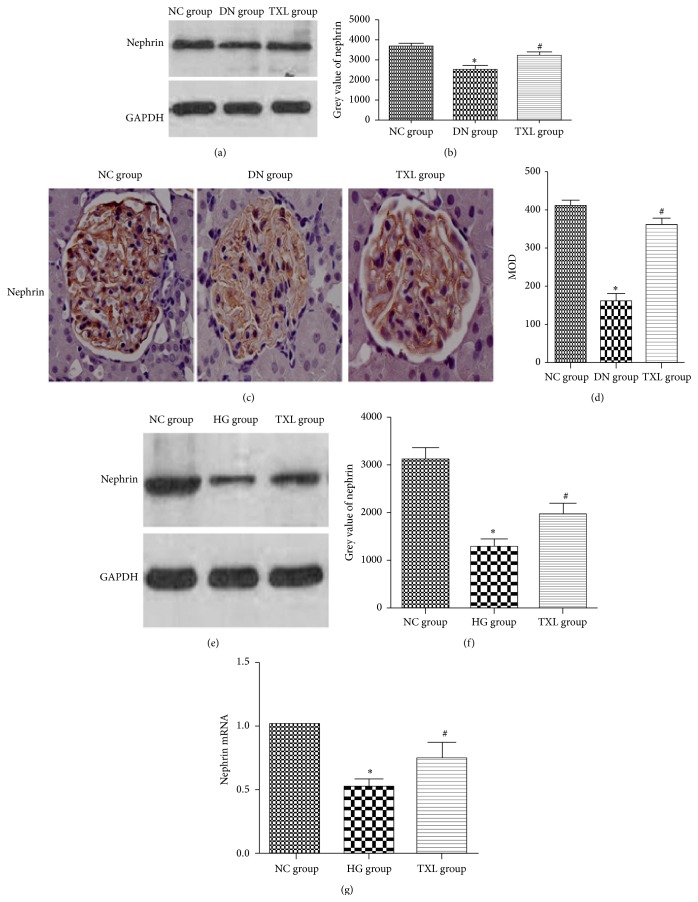
Effect of TXL on nephrin expression in diabetic rats and high glucose cultured podocyte. (a) Representative band of nephrin protein by western blot in rats. (b) Comparison of the grey value of nephrin protein in rats (*n* = 3). The result showed that nephrin protein was statistically significantly decreased in DN group compared with NC group (*P* < 0.05). Nephrin protein expression was significantly increased in TXL group compared with DN group (*P* < 0.05). (c) Representative immunohistochemical photograph for nephrin in vivo. Immunohistochemical results showed that nephrin protein expression was significantly decreased in DN group. (d) Comparison of mean optical density (MOD) of nephrin protein in rats. The result showed that nephrin protein was statistically significantly decreased in DN group compared with NC group (*P* < 0.05). Nephrin protein expression was significantly increased in TXL group compared with DN group (*P* < 0.05). (e) Representative band of nephrin protein by western blot in cultured podocyte. (f) Comparison of the grey value of nephrin protein in cultured podocyte (*n* = 3). The result showed that nephrin protein was statistically significantly decreased in HG group compared with NC group (*P* < 0.05). Nephrin protein expression was significantly increased in TXL group compared with HG group (*P* < 0.05). (g) Comparison of mRNA level of nephrin by RT-PCR in cultured podocyte (*n* = 3). The result showed that nephrin mRNA was statistically significantly decreased in HG group compared with NC group (*P* < 0.05). Nephrin mRNA expression was significantly increased in TXL group compared with HG group (*P* < 0.05). ^*∗*^ *P* < 0.05 versus NG. ^#^ *P* < 0.05 versus HG.

In our in vivo study, nephrin protein expression was detected by western blot and immunohistochemistry. Compared with NC group, nephrin expression was decreased in diabetic rats. TXL attenuated decreased nephrin expression in diabetic rats. Meanwhile, nephrin mRNA and protein expression were detected in cultured podocyte by RT-PCR and western blot. In accordance with our in vivo study, nephrin protein expression was significantly decreased in HG group compared with NC group (*P* < 0.05). Moreover, nephrin mRNA transcript level was also significantly decreased in high glucose cultured podocyte (*P* < 0.05). TXL reversed decreased nephrin protein expression and mRNA transcript level induced by high glucose (*P* < 0.05) (Figure 2).

### 3.3. TXL Inhibited the Activation of Notch Pathway In Vivo and In Vitro

**Figure 3:**
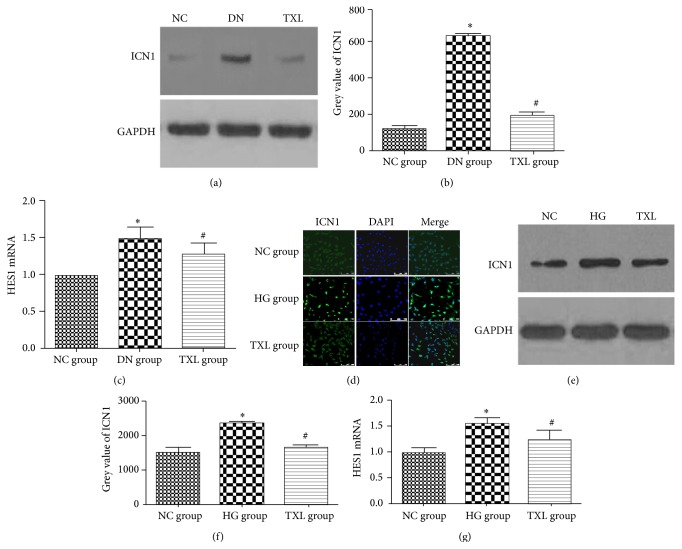
Effect of TXL on notch pathway in diabetic rats and high glucose cultured podocyte. (a) Representative band of ICN1 protein by western blot in rats. (b) Comparison of the grey value of ICN1 protein in rats (*n* = 3). The result showed that ICN1 protein was statistically significantly increased in DN group compared with NC group (*P* < 0.05). ICN1 protein expression was significantly decreased in TXL group compared with DN group (*P* < 0.05). (c) Comparison of mRNA level of HES1 by RT-PCR in rats (*n* = 3). The result showed that HES1 mRNA was statistically significantly increased in DN group compared with NC group (*P* < 0.05). HES1 mRNA expression was significantly decreased in TXL group compared with DN group (*P* < 0.05). (d) Representative photograph of ICN1 staining (green) and cell nucleus (DAPI blue). High glucose increased ICN1 expression and promoted ICN1 translocation from cytoplasm to nucleus, and TXL inhibited ICN1 expression and nuclear translocation. (e) Representative band of ICN1 protein by western blot in cultured podocyte. (f) Comparison of the grey value of ICN1 protein in cultured podocyte (*n* = 3). The result showed that ICN1 protein was statistically significantly increased in HG group compared with NC group (*P* < 0.05). ICN1 protein expression was significantly decreased in TXL group compared with HG group (*P* < 0.05). (g) Comparison of mRNA level of HES1 by RT-PCR in cultured podocyte (*n* = 3). The result showed that HES1 mRNA was statistically significantly increased in HG group compared with NC group (*P* < 0.05). HES1 mRNA expression was significantly decreased in TXL group compared with HG group (*P* < 0.05). ^*∗*^ *P* < 0.05 versus NG. ^#^ *P* < 0.05 versus HG.

As we know, the initial step in the activation of notch pathway is the proteolytic cleaving of the intracellular domain of notch1 by *γ*-secretase, with consequent release of ICN1 that translocates into the nucleus. HES1, a downstream target gene of ICN1, is an important component of notch pathway [24]. Herein, ICN1 and HES1 expression were detected in our in vivo and in vitro study. In our in vivo study, ICN1 and HES1 expression were detected by western blot and RT-PCR, respectively. Our results showed that ICN1 protein expression was increased in DN group. HES1 expression was increased in DN group compared with NC group. TXL decreased ICN1 and HES1 expression in diabetic rats. In our in vitro study, ICN1 expression was detected by immunofluorescence and western blot, and HES1 transcript level was detected by RT-PCR. Our immunofluorescence results showed that ICN1 was weakly distributed in the cytoplasm of podocyte in NC group. High glucose increased ICN1 expression and promoted ICN1 translocation from cytoplasm to nucleus. TXL inhibited ICN1 expression and nuclear translocation. ICN1 protein expression, detected by western blot, was increased in HG group compared with NC group (*P* < 0.05). TXL markedly decreased ICN1 protein expression induced by HG (*P* < 0.05). Moreover, high glucose significantly increased HES1 mRNA expression (*P* < 0.05). TXL markedly decreased HES1 mRNA expression induced by HG (*P* < 0.05) (Figure 3).

### 3.4. TXL Decreased Snail Expression In Vivo and In Vitro

**Figure 4:**
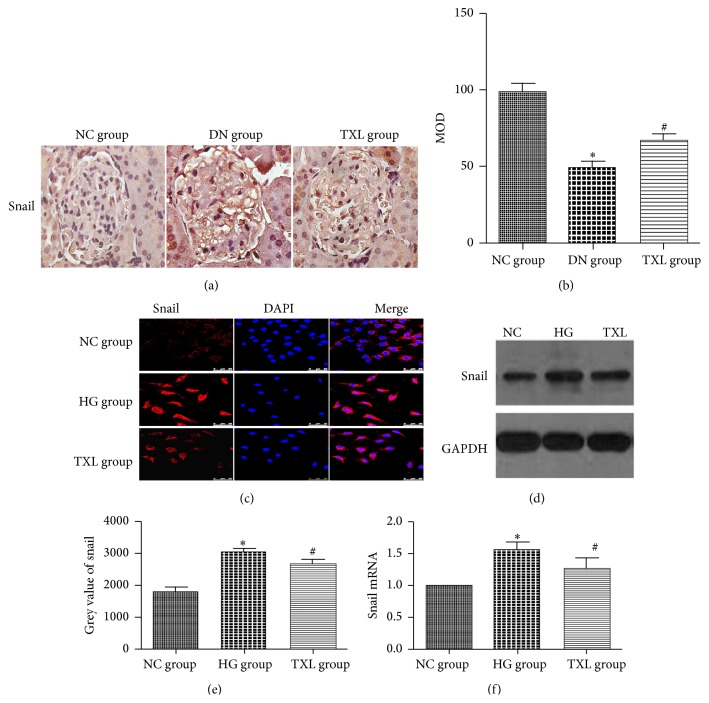
Effect of TXL on snail expression in diabetic rats and high glucose cultured podocyte. (a) Representative immunohistochemical photograph for snail in vivo. Immunohistochemical results showed that nephrin protein expression was significantly increased and translocated from cytoplasm to nucleus in DN group. TXL decreased snail protein expression and inhibited the translocation from cytoplasm to nucleus in diabetic rats. (b) Comparison of mean optical density (MOD) of snail protein in rats. The result showed that snail protein was statistically significantly decreased in DN group compared with NC group (*P* < 0.05). Snail protein expression was significantly increased in TXL group compared with DN group (*P* < 0.05). (c) Representative photograph of snail staining (red) and cell nucleus (DAPI blue). High glucose increased snail expression and promoted snail translocation from cytoplasm to nucleus, and TXL inhibited snail expression and nuclear translocation. (d) Representative band of snail protein by western blot in cultured podocyte. (e) Comparison of the grey value of snail protein in cultured podocyte (*n* = 3). The result showed that snail protein was statistically significantly increased in HG group compared with NC group (*P* < 0.05). Snail protein expression was significantly decreased in TXL group compared with HG group (*P* < 0.05). (f) Comparison of mRNA level of snail by RT-PCR in cultured podocyte (*n* = 3). The result showed that snail mRNA was statistically significantly increased in HG group compared with NC group (*P* < 0.05). Snail mRNA expression was significantly decreased in TXL group compared with HG group (*P* < 0.05). ^*∗*^ *P* < 0.05 versus NG. ^#^ *P* < 0.05 versus HG.

Snail, a DNA-binding molecule, is increased in injured podocyte. It has been demonstrated that snail directly decreased nephrin expression through binding the segment C1 of nephrin gene [25]. Moreover, HES1 can directly regulate snail expression in other cell lines [26]. Thus, snail acts as a bridge for decreased nephrin expression and activated notch pathway in podocyte in DN. Similarly, snail expression of renal cortex was detected by immunohistochemistry in our in vivo study. Our results showed that snail expression was increased in DN group compared with NC group. TXL decreased snail expression in diabetic rats. Next, the effect of TXL on snail was explored in cultured podocyte. Immunofluorescence results showed that snail weakly located in the cytoplasm of podocyte in NC group. In HG group, snail was transferred into the nucleus and its expression was increased. TXL inhibited snail expression and nuclear translocation. Moreover, snail protein and mRNA expression level, detected by western blot and RT-PCR, were significantly increased in HG group compared with NC group (*P* < 0.05). TXL markedly inhibited snail protein and mRNA expression induced by HG (*P* < 0.05) (Figure 4).

### 3.5. TXL Protected Podocyte from Injury In Vivo and In Vitro

**Figure 5:**
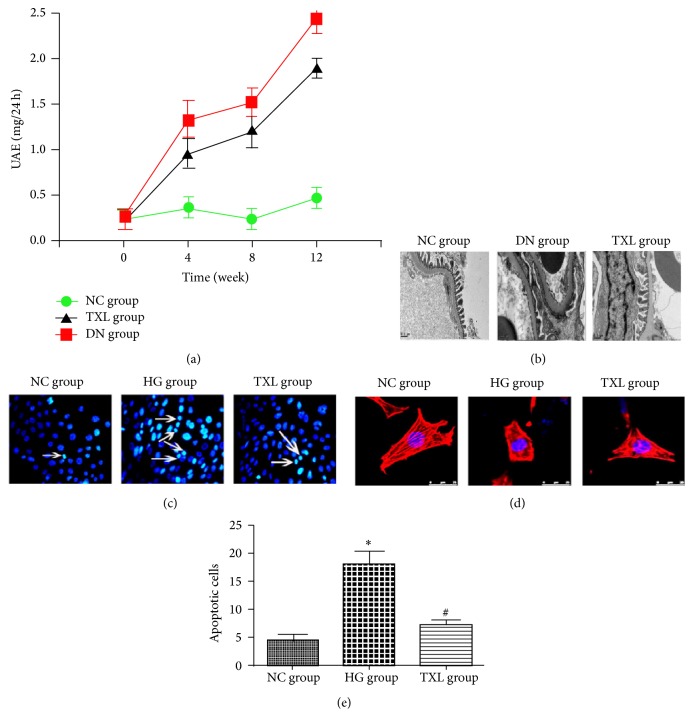
Effect of TXL on podocyte injury in diabetic rats and in high glucose cultured podocyte. (a) Comparison of UAE in three groups (*n* = 12). At the beginning of our study, UAE was not significantly different in three groups. At the end of 4, 8, and 12 weeks, UAE was increased in a time-dependent manner in DN group. Compared with DN group, UAE was decreased in TXL group at the end of 4, 8, and 12 weeks. (b) Representative photograph of podocyte foot process by electron microscopy. Compared with NC group, podocyte foot became fused and flattened in DN group. TXL significantly ameliorated podocyte foot effacement in diabetic rats. (c) Representative photograph of apoptotic cells by Hoechst 33258 staining (arrows indicate cell nuclear condensation). (d) Representative photograph of podocyte cytoskeleton by phalloidin staining. In NC group and MA group, podocyte cytoskeleton was normally arranged as long stress fiber-like bundles. In HG group, intracellular actin stress fibers were abolished and placed by cortical actin web, resulting in a polygonal cellular shape. (e) Comparison of apoptotic cells in three groups. The average number of apoptotic cells was assessed in 10 random fields per group. Compared with NC group, apoptotic cells were significantly increased in HG group (*P* < 0.05). Apoptotic cells were significantly decreased in TXL group compared with HG group (*P* < 0.05). ^*∗*^ *P* < 0.05 versus NG. ^#^ *P* < 0.05 versus HG.

Decreased nephrin expression has an intensive relationship with podocyte apoptosis and podocyte cytoskeleton change. The change of podocyte cytoskeleton can lead to podocyte foot process effacement and proteinuria. Herein, UAE, podocyte foot process, podocyte cytoskeleton, and podocyte apoptosis were detected in our in vivo and in vitro study. In our in vivo study, UAE was increased and podocyte foot process became fused and flattened in DN group. TXL significantly decreased UAE and alleviated podocyte foot effacement in diabetic rats. Our in vitro study showed that apoptotic cells were significantly increased in HG group compared with NC group (*P* < 0.05). Administration of TXL significantly decreased the number of apoptotic cells induced by high glucose (*P* < 0.05). In NC group, podocyte cytoskeleton was observed as parallel bundles of stress fibers. In HG group, intracellular actin stress fibers were abolished and placed by cortical actin web, resulting in a polygonal cellular shape. Moreover, TXL resumed intracellular actin stress fibers and maintained the normal cellular shape of high glucose cultured podocyte (Figure 5).

## 4. Discussion

Accumulate evidence has shown that podocyte loss and injury are an important mechanism of DN [2–4]. Nephrin is a signature molecule of podocyte [5]. It has been demonstrated that nephrin expression is significantly decreased in DN [6–9]. In accordance with previous studies, our study showed that nephrin expression was decreased in diabetic rats and in high glucose cultured podocyte. More importantly, TXL increased nephrin expression in vivo and in vitro.

Evidence is available that notch pathway is a common pathway of podocyte injury [10]. It has been demonstrated that the activation of notch pathway can downregulate nephrin expression in previous studies [11, 12]. Meanwhile, Gagliardini et al. demonstrate that persistent activation of notch1 and snail signaling in podocyte contributes to decreased nephrin expression in DN [13]. In our study, ICN1, HES1, and snail expression were increased in diabetic rats and in high glucose cultured podocyte, indicating the activation of notch/snail pathway. Moreover, the administration of TXL significantly inhibited the activation of notch/snail pathway in vivo and in vitro.

As we know, UAE is increased and podocyte foot becomes effaceable in DN. Moreover, previous studies have demonstrated that decreased nephrin expression can lead to podocyte apoptosis and podocyte cytoskeleton change [27–29]. Our study also found that UAE was increased and podocyte foot became effaceable in diabetic rats, accompanied with decreased nephrin expression. Meanwhile, HG significantly increased the number of apoptotic cells and induced the change of cytoskeleton in cultured podocyte in our in vitro study. As TXL alleviated the downregulation of nephrin in podocyte, the effect of TXL on podocyte injury in DN was explored in our study. Our results showed that administration of TXL significantly decreased UAE and ameliorated podocyte foot effacement in diabetic rats. Moreover, TXL significantly decreased the number of apoptotic cells and resumed abnormal cytoskeleton. Thus, we conclude that TXL has a protecting effect on podocyte injury in DN.

## 5. Conclusion

In conclusion, we speculated that TXL might have a pleiotropic effect on notch/snail pathway and nephrin expression both indirectly and directly, which may be a mechanism of protected effect on podocyte injury in DN. However, our study has some limitations and pitfalls. Although TXL has a pleiotropic effect on podocyte injury, the main therapeutic target of TXL is still unclear. Thus, further studies need to be performed.
